# What the Hospitals Do for the Public

**Published:** 1893-05-27

**Authors:** William S. Savory


### WHAT THE HOSPITALS DO FOR THE PUBLIC.

By Sir William S. Savory, Bart., F.R.S.

Sir William Savory, in replying to the toast of "The Medical Staff" at the Great Northern Central Hospital dinner, made an excellent speech, and we are indebted to him for being able to publish it in full:—

You have paid me a great compliment in allowing my name to be associated with this toast, but although I very gladly respond to it I must confess myself somewhat of a pretender to the honour of being on the surgical staff, for my position there is really only a formal one. I have no share in the work, and so I can take none of the credit which belongs to it. But now there is this advantage in standing on the outside: I can speak with less reserve of what the hospital is and does.

The praise of hospitals is a well-worn theme. We are familiar with the acknowledgment that they are conspicuous among the charities of London, and in the help they give are free from the reproach to which so many other charities are open. A poor man struck down by accident at his work, or a woman or a child by illness, is surely beyond all cavil a fit subject for charitable help. But after all, what does a hospital like this mean? To call it simply a great house of charity is surely a very inadequate expression of its work, for it might fulfil its mission without censure in this respect, and yet be very different from what it is. The kind of medical and surgical help which patients receive in hospitals is due to resources and conditions which cannot be commanded elsewhere. The kind of medicine and surgery means that which is the outcome of the advantages derived from being in the midst of the best and highest contemporary work, the result of the keenest struggle with honourable rivals of the foremost rank, in the face of friendly but unsparing criticism, and in the wholesome light of full publicity. Nor is this all. Patients here have not only the advantage of all this, but in thus receiving help they are protected from evils perhaps more important still. They are protected from quackery, fraud, and imposture, against which without its walls neither wealth nor rank, nor any position is secure; for the worst forms of quackery, the offspring of ignorance and cupidity, find their most ready victims among the prosperous and powerful.

And not the poor only who come within these walls, but the whole country is indebted to them for medical and surgical help, for it is really only through hospitals that men can be properly trained and educated to become good physicians and surgeons. And further, hospitals are almost beyond comparison the chief means by which medicine and surgery are advanced. They are the supreme seats of their culture, in which not only is the knowledge of the science and art promoted, but the character of those who practice them is formed, maintained, and raised. I venture to say whatever may be the claims of medicine and surgery in their intellectual and moral aspect, they largely, very largely, owe to hospitals. Need I say more? That His Royal Highness the Duke of York has been pleased to preside over us to day is at once a proof of worthiness of the claims of this hospital, and a guarantee of its future prosperity and progress. And, indeed, it is a source of the heartiest congratulation to us all—to the profession and the public—that the deep personal interest which our Queen and the Prince of Wales have always taken in our hospitals and in the welfare of the sick is evinced by His Royal Highness, in whose presence we rejoice this evening.

